# Enhancing the Interfacial Strength of Carbon Fiber/Poly(ether ether ketone) Hybrid Composites by Plasma Treatments

**DOI:** 10.3390/polym11050753

**Published:** 2019-04-28

**Authors:** Chunrui Lu, Si Qiu, Xue Lu, Jian Wang, Lin Xiao, Ting Zheng, Xiaodong Wang, Dongxing Zhang

**Affiliations:** 1 School of Materials Science and Engineering, Harbin Institute of Technology, Harbin 150001, China; luxue2014hgd@126.com (X.L.); wj8958@163.com (J.W.); linxiao0501@gmail.com (L.X.); zthappy1127@gmail.com (T.Z.); 2 School of Chemistry and Materials Engineering, Huizhou university, Huizhou 516007, China; qiusi250@gmail.com; 3 College of Material Science and Chemical Engineering, Harbin Engineering University, Harbin 150001, China

**Keywords:** polymer (textile) fibers, hybrid, interface, mechanical properties

## Abstract

As a promising alternative to traditional prepreg, carbon fiber/poly(ether ether ketone) (CF/PEEK) hybrid composites have attracted wide public interest for their flexibility and conformability. However, modification methods focused on the hybrid premix have not been previously studied. In the present work, the interfacial strength of the hybrid composite was improved by treating the carbon and PEEK fibers together in a radiofrequency (RF) plasma containing one of the following gases to achieve surface activation: air, Ar, or Ar–air. After plasma treatment, the increased roughness of CF and the grafted chemical groups of CFs and PEEK fibers were propitious to the mechanical interlocking and interfacial strength. Significant interfacial shear strength (IFSS) enhancement was achieved after Ar 1 min, air 1 min plasma treatment. This study offers an alternative method for improving the interfacial properties of CF/PEEK composites by focusing on the boundary layer and modifying and controlling the fiber–matrix interface.

## 1. Introduction

With the increasing demand for high-performance composites for aerospace, automobile, and other structural applications in engineering, great efforts have been devoted to the study and development of fiber-reinforced thermoplastic composites [1,2,3]. Among these, carbon fiber/poly(ether ether ketone) (CF/PEEK) composites have attracted wide attention for the superior properties of their semicrystalline PEEK matrix, including their high service temperature, outstanding mechanical properties, and dimensional stability [4,5,6]. However, the high melting viscosity and the difficulty of infiltrating carbon fibers during CF/PEEK composite fabrication must be addressed. Many studies have investigated the 2-D and 3-D hybrid CF/PEEK premixes, a subject that has attracted wide attention [4,7]. The resulting mechanical properties of CF/PEEK depend mainly on the interfacial property, which plays an important role in stress transfer between the reinforcement and the matrix [8,9,10]. The physical state and chemical structure of CFs and PEEK fiber determine the interfacial strength [11]. Hence, the low interfacial bonding strength between CF and PEEK fiber matrices due to the intrinsically smooth, hydrophobic, and chemically inert CF surface is a problem that must be addressed. Recently, significant scientific efforts have focused on the surface modification of CF to improve the interfacial properties of composites by electrochemical [12] and chemical treatments [13], plasma etching [6], and the sizing technique [14]. However, some of these methods enhance the interfacial adhesion strength of the composites at the expense of damaging the inherent structure of CFs, leading to a decrease in the strength of the CF.

In recent years, an increasing number of researchers have focused on the plasma modification technology, mainly for its advantages compared with other methods, such as high efficiency and surface limitation [15,16]. Plasma modification has high efficiency and can achieve satisfactory results in a few seconds to a few minutes [17]. The effect of plasma treatment is limited to a thin layer on the material’s surface, thereby only changing the physical and chemical properties of the surface within tens to hundreds of nanometers without affecting the mechanical properties of the fiber’s inherent structure. The etching depth can also be adjusted depending on the time of treatment [18]. Plasma treatment is a dry and physiological process, which does not use or produce chemical substances and therefore does not cause environmental pollution. In this treatment, the reaction atmosphere comprises different types of gases [19]. The use of different gas combinations in different proportions can diversify the modification reaction so as to address the actual demand [20,21]. It is necessary to introduce specific reaction groups and elements on the surface of materials according to different matrices. These findings indicate that the plasma modification technology has broad application prospects for fiber modification [22,23].

Plasma treatment has been widely applied for CF modifications using high-energy particles, such as electrons and ions, to cause oxidation and candles on the surface of fibers. Hence, this method can achieve the following: remove the weak interfacial layer on CF surfaces; increase the polarity and roughness of the surface; enhance the chemical bonding and physical chimerism between fibers and resin matrices; and improve the interfacial strength of composites [22]. However, thermoplastic composites are usually processed from polymers in the form of film, pellet, and powder. These polymers are extraordinarily large in size compared with the modified scale of CFs, resulting in limitations of appraisal for the modification effects. Therefore, composites molded from CFs and PEEK fibers can potentially improve the interfacial properties. Furthermore, modification methods focusing on woven fabrics, including the reinforcement and matrix simultaneously, will be meaningful for research and application.

In our study, plasma treatment by air, Ar, or Ar–air was applied. The physicochemical properties of CFs and PEEK fibers before and after plasma treatment were investigated. Surface morphology changes were characterized by scanning electron microscopy (SEM). The chemical structures and specific properties of CFs were studied by Raman spectra and single-fiber tensile tests. Fourier transform infrared spectroscopy (FTIR) and differential scanning calorimetry/thermogravimetric analysis (DSC/TGA) were carried out to study PEEK fibers and compare their structure and properties before and after plasma treatment. Element content and surface group composition were studied by X-ray photoelectron spectroscopy (XPS). The microdroplet test was used to assess the interfacial shear strength (IFSS). The results were then analyzed.

## 2. Materials and Methods

### 2.1. Materials

The commercial polyacrylonitrile (PAN)-based carbon fiber tows (T300B-1000-50B, 1.76 g/cm^2^) with a mean diameter of 7.5 μm were supplied by Toray Industries Inc. (Tokyo, Japan). PEEK fibers (850D/144F), with an average diameter of 27 μm, was purchased from Changzhou Co–Win Novel Materials Co, Ltd. (Changzhou, China). The fibers were all cleaned using ethyl alcohol and acetone. They were then dried in a vacuum oven at 80 °C for 1 h, and the dust, grease, and chemical residues were removed without altering the sizing on fibers.

### 2.2. Plasma Treatments

**Figure 1:**
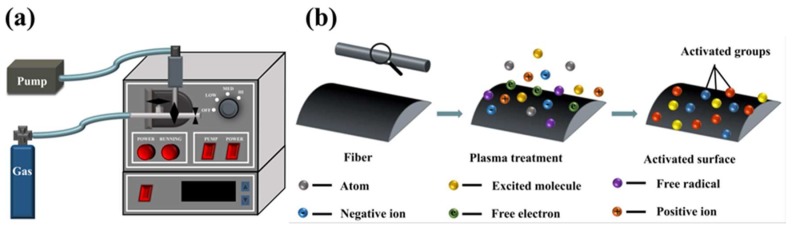
Diagram of the plasma treatment equipment: (**a**) plasma treatment equipment; (**b**) mechanism of surface plasma treatment.

The plasma treatment process was performed using a PDC36G plasma cleaner with adjustable radiofrequency (RF) power supply (purchased from Hefei Kejing Materials Technology Co., Ltd, Hefei, China), which is usually used to clean nanoscale organic pollutants on substrates or crystals. This equipment is composed of a vacuum chamber, a needle valve, a resistance vacuum gauge, a vacuum pump, and a radio source (Figure 1a). The plasma treatment was conducted in an inductively coupled radio frequency (13.56 MHz) plasma reactor with a high-power mode (18 W). Gas was fed into the vacuum chamber at a flow rate of about 6–8 standard cubic centimeters per minute (SCCM). The operational pressure was set at 1 Torr. Dozens of carbon and PEEK fiber tows were carefully fixed across a glass frame and treated using air plasma (2 min), Ar plasma (2 min), and Ar–air plasma (Ar 15 s, air 15 s; Ar 1 min, air 1 min; Ar 5 min, air 5 min) for different times. Samples treated by Ar–air were labeled as AA15, AA1, and AA5. After the samples were subjected to different treatments, they were stored in a desiccator at room temperature until they were analyzed by SEM, Raman spectroscopy, DSC/TGA, and XPS.

By definition, plasma is a neutrally ionized gas on a spatial scale, containing an equal number of negative and positive ions, a small percentage of free radicals, a few parts of atoms, excited molecules and free electrons, and a large amount of extremely energetic vacuum ultraviolet light (Figure 1b). The basic theoretical foundation in plasma treatment of materials is free-radical chemistry. Gases are activated and dissociated using a radio source, which acts via electron bombardment and photochemical processes to create a high density of gas-phase free radicals. Among them, free radicals in electronically excited states carry a great deal of energy that is sufficient to break any organic bond. This results in the abstraction of atoms or molecular fragments that can react further in the plasma to form volatile species. In addition, abstraction causes a progressive ablation of organic surfaces and the formation of residual free radicals on that surface. These can either react with themselves, resulting in surface cross-linking, or react with the plasma gas, even ground state molecules, to form new chemical species on the surface. If the gas used to produce plasma is inert (like argon), after treatment, the surface would contain a large number of stable radicals that can further react until exposed to reactive gases. Thus, plasma treatments are effective for breaking and creating bonds on the surface of polymers or organically contaminated substrates [23].

### 2.3. Characterizations

#### 2.3.1. Surface Morphology

Scanning electron microscope (Merlin Compact, ZEISS, Dresden, Germany) measurements were used to observe surface topography and provide a general analysis for the material before and after modification.

#### 2.3.2. Raman Spectroscopy

Raman spectroscopy is an effective experimental technique used to determine information on carbon fiber microstructure. Raman spectroscopy data were collected using a INVIA010410 Laser micro confocal Raman spectrometer (Renishaw, London, UK) with a wavelength and laser beam size of 514.5 nm of argon laser (green) and ~2 µm diameter. A 500× magnification microscope was used to select a point of focus for the laser beam spot on the fiber’s surface. The scanning range was set at 500–4000 cm^−1^ to obtain the first- and second-order Raman bands for CF. The band intensity, band width, and band position were obtained from the first-order Raman spectra using a Gauss curve-fitting procedure.

#### 2.3.3. Single-Fiber Tensile Test

**Figure 2:**
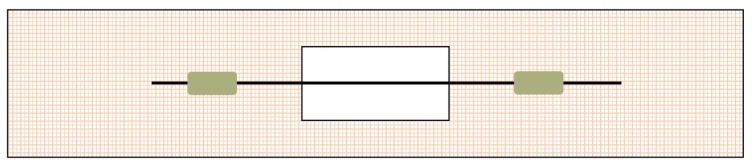
Schematic diagram of monofilament tensile specimen.

Single-fiber tensile tests were performed on CFs treated with different plasma conditions using a Sintech universal testing machine following the ASTM D3379 standard test method. The test sample was obtained by cutting a section of 12 cm fiber, pulling out to get the monofilament, and then fixing it on the paper frame using glue and tensile force to keep it straight, as shown in Figure 2. During the test, the paper frame was fixed vertically in the upper and lower clamps of the electronic universal testing machine. The paper frame was cut off before loading. After debugging the instrument, the load was applied, and the peak load was recorded automatically by the testing machine. In this experiment, paper frame gauge was selected as 2 cm.

#### 2.3.4. Fourier Transform Infrared Spectra (FTIR)

The chemical structure of PEEK before and after plasma treatment was examined based on the Nicolet iS50 Fourier transform infrared spectra (Thermo Fisher, Waltham, MA, USA), using the KBr pellet method at 500–4000 cm^−1^ wavelength.

#### 2.3.5. Thermal Analyses

Differential scanning calorimetry measurements were performed in order to study the effect of plasma treatment on the melting point of PEEK fibers using STA449F3 synchronous thermal analyzer (NETZSCH, Selb, Germany). The fibrous PEEK, cut into small length sizes with ~10 mg weight, were thermally cycled between 25 °C and 400 °C twice at a constant heating/cooling rate of 10 °C min^−1^ in a nitrogen atmosphere. The thermogravimetric analysis tests for PEEK fibers (~10–15 mg) were conducted in a nitrogen atmosphere from 25 °C to 800 °C at a heating rate of 10 °C min^−1^ to study changes in the PEEK fiber’s thermal stability induced by plasma treatment.

#### 2.3.6. X-Ray Photoelectron Spectroscopy (XPS)

XPS analyses were conducted using the ESCALAB 250Xi (Thermo Fisher, Waltham, MA, USA) to study the surface elements and groups of CFs and PEEK fibers after plasma treatment. XPS spectra were obtained using an Al Ka (*hv* = 1486.6 eV) monochromated X-ray source at 15 kV and 150 W. The pass energy and energy step were 20.0 eV and 0.05 eV, respectively. A nonlinear least-square curve-fitting program (XPSPEAK software 4.1, Raymund W.M.Kwork) was used to deconvolve the XPS data. To compensate for the charging effects, all spectra were calibrated with graphitic carbon as the reference at a binding energy (BE) of 284.6 eV. The spectra were deconvolved by subtracting a Shirley + linear background, and a Lorentzian–Gaussian (GL = 20%) mixed function. The surface chemical composition was calculated from the peak areas of relevant spectra.

### 2.4. Interfacial Shear Strength

**Figure 3:**
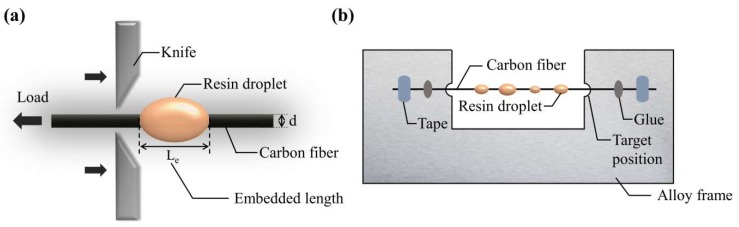
Illustrations for the microdroplet test: (**a**) the process of microdroplet test, (**b**) the scheme of the sample.

The microdroplet test was conducted on HM410 evaluation equipment, which determines the interfacial property of composite material (Tohei Sangyo Co., Ltd, Tokyo, Japan)—to directly test the interfacial sheer strength (IFSS) between CF and PEEK. The process is illustrated in Figure 3a. In this experiment, each microdroplet was clamped by two steel blades and pulled to separate with the fiber at a constant speed of 0.1 mm/min. The microdroplet test specimens were prepared on alloy frames (30 mm × 70 mm) with the free fiber length ~30 mm between the frames. Both sides of the CF single fiber were firmly fixed on the alloy frame, and some oval PEEK droplets obtained by melting the PEEK fibers were formed on a CF monofilament, as shown in Figure 3b. To obtain perfectly shaped PEEK droplets, we started by laying an alloy frame fixed with filament on a closed heating platform at 375 °C for 10 min and sequentially melted one side of the PEEK tows while holding the other side to form fine PEEK fiber to cross the monofilament. This process was repeated for a sufficient number of times. The single fiber composite specimens were then transferred to a vacuum oven at 375 °C for 5 min to reheat the composite specimens in order to obtain perfectly shaped microdroplets and wetted interfaces.

## 3. Results and Discussion

### 3.1. Morphology of CF and PEEK Fiber

**Figure 4:**
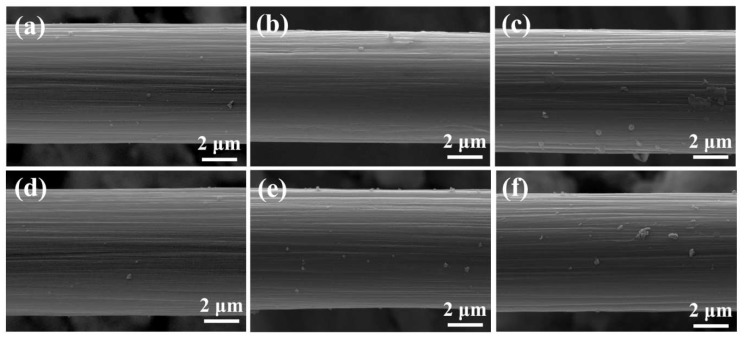
SEM images of carbon fibers (CFs) at different plasma treatment times: (**a**) untreated CF, (**b**) CF–AA15 (Ar 15 s, air 15 s), (**c**) CF–air, (**d**) CF–Ar, (**e**) CF–AA1 (Ar 1 min, air 1 min), and (**f**) CF–AA5 (Ar 5 min, air 5 min).

The surface morphology of CFs at different plasma treatment times are shown in Figure 4a–f. The data demonstrate the presence of clear ridges and striations on the surface of CF parallel to the fiber’s axial direction. These formations are a result of the carbon fiber manufacturing process, such as stretching and sizing, and the gullies provide an excellent condition for infiltration and adhesion of polymers. Within a short plasma treatment time like 30 s (the total plasma treatment time), the patches on the surface of untreated carbon fibers almost disappeared due to the etching action and cleaning effect of the plasma treatment. Patch removal from the surface ameliorated the potential weak boundary condition in fiber-reinforced polymer composites, thus improving the connection between the fiber and the matrix. However, the modification effects within the same time varied depending on the type of gas (shown in Figure 4c,d), owing to the different energy density of the plasma. Plasma energy density is related to treatment time, plasma power, and area of the electrodes [24]. In this condition, the number and size of samples treated by air plasma with the largest plasma power was the most complicated among CF–air, CF–Ar, and CF–AA1, whereas CF–Ar was the simplest [24]. Increasing plasma treatment time was associated with increased complexity and generation of embossments on the surface. The number and size of bulges increased gradually with prolonged treatment time. This phenomenon can be attributed to the bombardment of high-energy particles or free radicals in the plasma on the carbon fiber surface. Plasma sputtering can destroy the intrinsic chains and surface groups of fiber, and some atoms would be knocked off at the same time [25,26,27]. Prolonged plasma treatment time provides a favorable condition, allowing the accumulation of these atoms and chains to form embossments.

**Figure 5:**
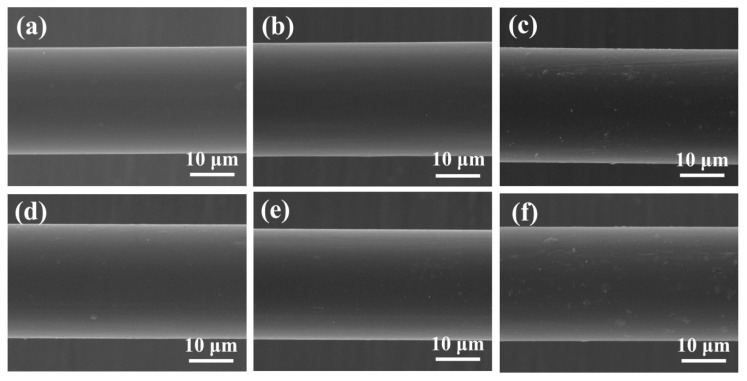
SEM images of poly(ether ether ketone) (PEEK) fibers at different plasma treatment times: (**a**) untreated PEEK fiber, (**b**) PEEK–AA15, (**c**) PEEK–air, (**d**) PEEK–Ar, (**e**) PEEK–AA1, and (**f**) PEEK–AA5.

Typical SEM images of the PEEK fibers before and after plasma treatment are shown in Figure 5a–f. The images clearly demonstrate that the surface of untreated PEEK fibers was relatively smooth and uniform without major irregularities. However, several patches existed on the surface of untreated PEEK fibers, which were not very obvious based on the fact that we could only find little traces in a comprehensive SEM analysis. These could be eliminated under the etching action and the cleaning effect after 30 s of plasma treatment. After a short plasma treatment time, e.g., 2 min, the surface of PEEK remained smooth with several obvious patches resulting from the high-energy particles or free radical bombardment on the surface, while the morphology of PEEK fibers changed according to the energy density of different plasma gases. As with CF, PEEK fibers treated with air plasma were rougher and displayed more patches than PEEK–Ar and PEEK–AA1. Increasing plasma treatment time resulted in an increase in the quantity and size of patches [28].

These data show that plasma treatment etches the surface of CFs and PEEK fibers and significantly changes the surface morphology. Although the etching depth is determined by time and instrument, the etching effect closely corresponds with the type of plasma gas. Prolonging plasma treatment time causes outstanding etching, resulting in a more complex surface morphology with increased surface roughness for both CFs and PEEK fibers [29]. Certainly, long treatment times influence the intrinsic properties of fibers, such as the mechanical properties of CFs and thermal properties of PEEK fibers, which are important during the molding process and application of CF/PEEK composites. Hence, further tests were performed, and discussions focusing on these particular questions are explained in the next section of this article. The complex surface morphology and increased roughness of CFs provide a large specific area for CF and PEEK bonding and thus enhance the mechanical intercalation between CF and PEEK fiber resins, which is an important factor for improving the interfacial adhesion between fibers and resins.

### 3.2. Structure and Mechanical Property Analysis of CFs

#### 3.2.1. Structure Analysis

Graphitic carbon and other sp^2^-bonded amorphous carbons possess strong Raman scatter despite their intense optical absorption. A Raman peak at ~1585 cm^−1^ is typical of bulk crystalline graphite, called the G band (ordered or graphitic). This peak represents the basic vibration mode of graphite crystal, and its intensity is closely related to the crystal size. The Raman peak at 1360 cm^−1^ originates from vibrations of the edge of graphite carbon and is called the D band, which represents disordered carbon. Thus, *R*-the ratio of D and G bands (*I_D_/I_G_*) is usually calculated and regarded as a measure of the size of graphite crystallites and the amount of amorphous carbon phase [30,31,32]. In our study, *R* values were obtained from the ratio of curve areas at ~1360 cm^−1^ and 1585 cm^−1^, and the crystalline size *L_a_* was calculated using the following relationship:

(1) $$L_{a}=C/R=C/\left(I_{D}/I_{G}\right)$$

where *C* = 44 Å.

**Figure 6:**
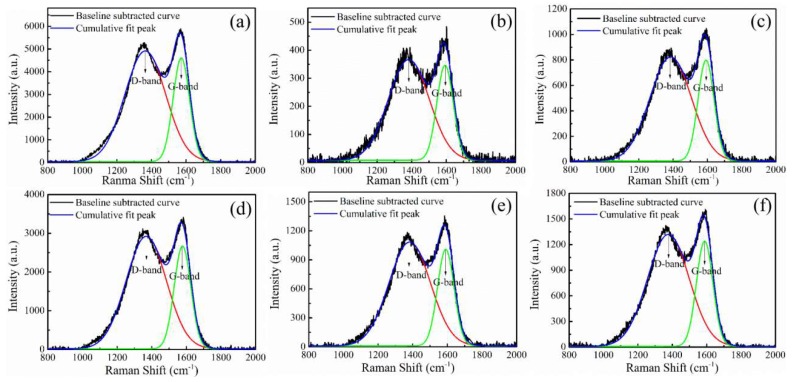
Raman spectra and their cumulative fit peaks for CFs before and after different plasma treatments: (**a**) untreated CF, (**b**) CF–air, (**c**) CF–Ar, (**d**) CF–AA15, (**e**) CF–AA1, and (**f**) CF–AA5.

The Raman spectra and its cumulative fit peak for CF before and after different plasma treatments are shown in Figure 6. The corresponding *R* and *L_a_* of untreated and treated carbon fibers obtained from Figure 6 are listed in Table 1. The data show that the value of *R* and *L_a_* did not significantly change with different plasma treatments and times. Thus, it can be concluded that plasma treatment might not change the crystalline or graphitic structure of carbon fibers because plasma treatments can only influence surfaces on a nanometer range.

#### 3.2.2. Tensile Strength of Carbon Fibers

**Figure 7:**
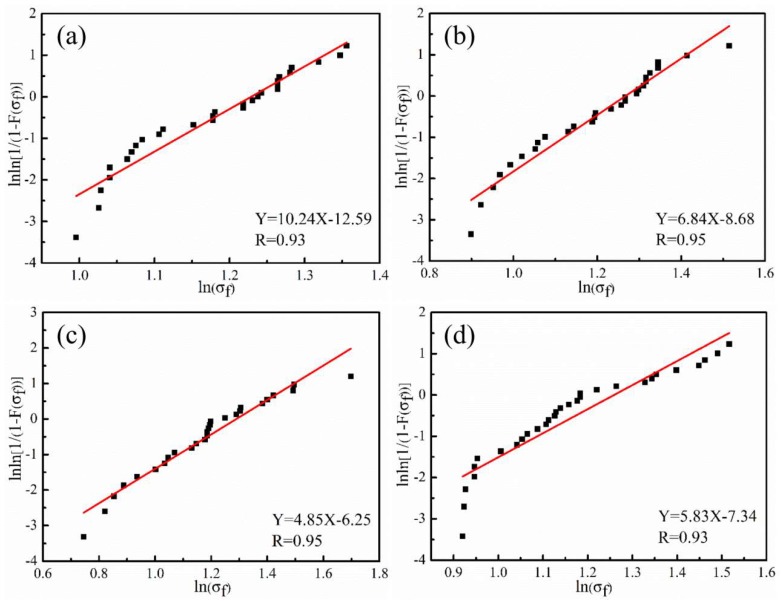
Weibull distribution curves of tensile strength for CF monofilaments under different plasma treatments: (**a**) untreated CF, (**b**) CF–AA15, (**c**) CF–AA1, and (**d**) CF–AA5.

As the etching effect depends on the plasma treatment time and equipment, we focused on the tensile strength of CFs treated for different times. The Weibull plots and fitted straight lines for single-fiber tensile tests are presented in Figure 7. The parameters required to obtain a fitted straight line, including scale parameter of unit fiber length ratio (σ_0_), Gamma function (Γ(σ_0_)), slope B, and intercept A of the straight line, are listed in Table 2. Based on the test results, the single-fiber tensile strength of untreated CFs was 3.25 GPa, and the value changed slightly during the tested plasma treatment times. This result indicates that a 10 min plasma treatment time merely affects the CF surface and causes little damage to their essential structures. However, as time extends, the etching depth increases, and damage to the intrinsic quality of CFs is inevitable [32]. Therefore, when treating CFs using plasma, its effect on the strength of bulk fibers should be considered and studied.

### 3.3. Chemical Structure and Thermal Property Analysis of PEEK Fibers

#### 3.3.1. Chemical Structure of PEEK Fiber

**Figure 8:**
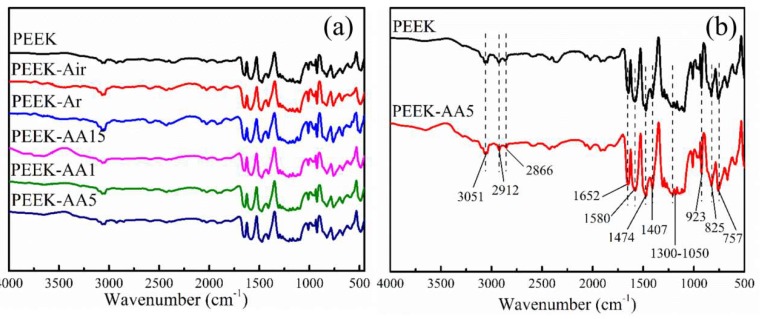
Selected FTIR spectra of PEEK fibers and PEEK–AA5: (**a**) untreated PEEK and (**b**) PEEK–AA5.

We used FTIR spectroscopy to investigate PEEK fibers before and after plasma treatment. The wide spectra from 4000 to 500 cm^−1^ for PEEK and different plasma-treated PEEK are shown in Figure 8a. In addition, PEEK and PEEK–AA5 with peak positions [32,33,34] are separately compared for detailed analyses in Figure 8b, with peak assignments provided in Table 3. Wavenumber values are shown for both spectra, and they were identified by reference to the absorbance bands shown in Table 3. FTIR measurements showed subtle distinctions between PEEK fibers under specific plasma conditions, even though significant peak data were presented. This could possibly be attributed to the technique’s 1–2 μm testing depth. Furthermore, the low sensitivity of FTIR impedes its application for determining trace components. As reported by the producer, the rate of surface etching is 20 nm/min at “Hi” for this plasma treatment equipment. In our test, the maximum treatment time was 10 min, and thus the etching depth was ~200 nm, which is far from the testing depth of 1–2 μm. This was still regarded as an effective result as it suggests that plasma treatment is aimed at surface modification, and the effects are therefore exclusively surface effects.

#### 3.3.2. Thermal Property Analysis

**Figure 9:**
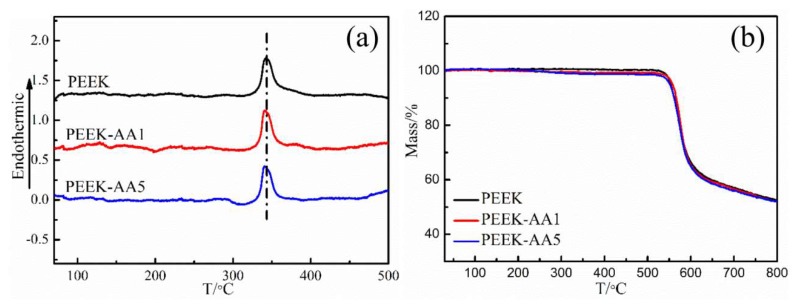
Differential scanning calorimetry (DSC) and thermogravimetric analysis (TGA) curves for PEEK fibers and plasma-treated PEEK fibers: (**a**) DSC curve and (**b**) TGA curve.

To study the effect of plasma treatments on the thermal properties of PEEK fibers, DSC/TGA was applied to test the samples’ glass transition temperature (*T*_g_) and thermal stability. DSC and TG curves of PEEK fibers treated with plasma for different lengths of time are shown in Figure 9. The data show that the glass transition temperature *T*_g_ of PEEK at 343 °C did not change after different durations of plasma treatment. Figure 9b shows that PEEK fibers and modified PEEK fibers began to lose weight at 525 °C, and the mass loss rate reached the maximum at 600 °C. Meanwhile, the thermogravimetric curves of PEEK fibers changed slightly, indicating that PEEK fiber modification using plasma treatments do not affect the thermal properties of PEEK. In this study, we introduced the etching and surface modification effects, which are both devoted to nanoscale depths of the surface material, making it difficult to influence the permanent thermal properties of PEEK fibers [35].

### 3.4. Surface Chemical Analyses

Wide-scan spectra with binding energy ranging from 0 to 1300 eV was obtained to identify surface elements, and the detailed narrow-scan spectra C1s and O1s were quantitatively analyzed to identify the chemical groups. C, O, and N contents on CFs and PEEK fibers before and after plasma modifications are shown in Table 4. The major constituents on CF and PEEK were carbon and oxygen. The appearance of nitrogen was due to the plasma modifications including air, Ar, and Ar–air. The proportion of oxygen increased after plasma treatment, both in CFs and PEEK fibers. In addition, the ratio of oxygen increased with prolonged plasma treatment time of 10 min. Plasma treatments by Ar–air increased the oxygen content compared with air and Ar treatments performed for the same amount of time (2 min). This can be explained by the reaction between the grafted active sites provided by Ar and air plasma [36].

**Figure 10:**
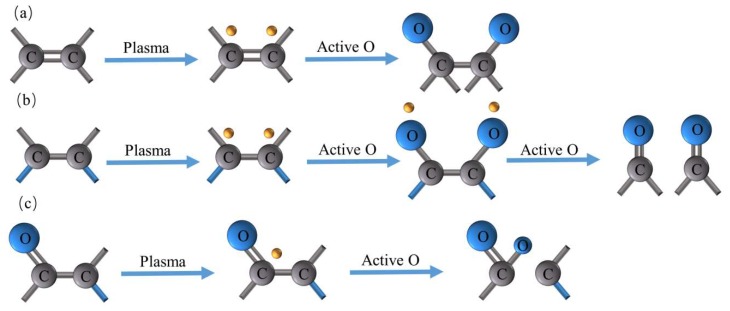
Possible mechanism of surface group oxidation by plasma treatment: (**a**) generation of C–O bonds, (**b**) generation of C=O bonds, (**c**) generation of O–C=O bonds.

The bonding energies corresponding to the groups that deconvolve from C1s of CFs and PEEK fibers and their concentrations are listed in Table 5. The C1s peak was fitted to four line shapes with binding energies at 284.0–284.3, 284.6–286.8, 285.6–286.1, 287.7–288.0, and 289.9–290.5 eV, corresponding to sp^2^-hybridized graphite carbon atoms (C=C), sp^3^-hybridized carbon atoms (C–C), C–O, –C=O, and O–C=O, respectively [36,37]. The proportion of C1 and C2 peaks for CF decreased after plasma treatment. For most samples, the proportion of C3, C4, and C5 increased by plasma modification to different extents depending on the types and times, except the C3 peak of CF–AA5, which showed an opposite trend. These changes indicated that the sp^2^ and sp^3^ carbon atoms were activated, damaged, and replaced with new oxygen on the surface during plasma treatment. Therefore, various oxidative reactions induced by plasma were predicted to occur. As reported, oxygen and argon plasma were able to create free radicals that coupled with active species in the plasma environment [38]. Due to the active C=C bond, it was more susceptible to plasma attack, and radicals were generated on the dissociated C=C bond for further reaction with active oxygen atoms. This was the reason for the decrease in the proportion of C=C bond after plasma treatment. Through this process, C–O bond was created, as illustrated in Figure 10a, while the new C=O bond was formed from oxygen radicals on the C–C bond, as shown in Figure 10b. The formation of O–C=O bonds was generated by C=O bond through combining radicals on the C=O bond with active oxygen atoms, as displayed in Figure 10c. However, the three presented reactions occurred throughout the plasma treatment process, and different bonds were activated or generated at the same time along with the changes in the element ratio. Hence, it is hard to describe the effect by the change of one group. Thus, the polar ratio combined with elemental proportion were introduced to study the effects caused by type and time of plasma treatment. Compared with air and Ar plasma treatments, CFs treated sequentially by Ar and air gained the largest number of polar groups. Proportions of polar peaks for CF–air, CF–Ar and CF–AA1 were close to each other, but the oxygen content of AA1 was larger than the other two for the fast graft effect after activation by Ar, indicating the better activation capacity of Ar–air. Prolonged treatment times increased oxygen content but not polar group content. Ratios in Table 5 were defined as the product of polar/nonpolar and effective element contents, including oxygen and nitrogen. Hence, we can conclude that samples treated with Ar–air gain the most effective group sites, which provide the most favorable conditions for interfacial adhesion. In addition, these methods are designed for hybrid CF/PEEK pre-prepreg; thus, CFs and PEEK fibers should be modified simultaneously. The CF results highlight the optimization conditions. The XPS study for PEEK fibers focused on the samples treated by Ar–air. For PEEK fibers, group concentration changes were more obvious. C3, C4, and C5 increased, while C1 and C2 decreased. However, the CFs and PEEK fiber group contents showed different degrees of increase or decrease. It can still be concluded that the polar oxygen groups were grafted onto CF and PEEK fiber surfaces by plasma treatment, but they varied with gas type and time. Furthermore, increasing polar contents on CFs and PEEK fibers probably supplied reactive sites for interfacial interaction between CFs and PEEK fibers.

### 3.5. Interface Shear Strength

**Figure 11:**
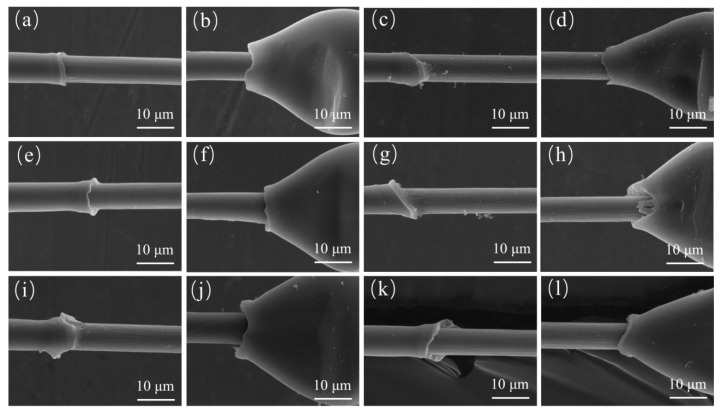
SEM images of samples after microdroplet test. (**a**) The beginning of CF/PEEK, (**b**) the ending of CF/PEEK, (**c**) the beginning of CF/PEEK–Air, (**d**) the ending of CF/PEEK–Air, (**e**) the beginning of CF/PEEK–Ar, (**f**) the ending of CF/PEEK–Ar, (**g**) the beginning of CF/PEEK–AA15, (**h**) the ending of CF/PEEK–AA15, (**i**) the beginning of CF/PEEK–AA1, (**j**) the ending of CF/PEEK–AA1, (**k**) the beginning of CF/PEEK–AA5, (**l**) the ending of CF/PEEK–AA5.

IFSS before and after plasma treatment was determined using the microdroplet test, and the destruction are shown in Figure 11. For untreated CF/PEEK system, there was not much retained matrix, but after plasma treatments, the retained matrix increased. The IFSS results are listed in Table 6. The IFSS of untreated CF/PEEK composites was 42.36 ± 4.23 MPa and increased to a maximum of 59.73 ± 3.88 MPa after plasma treatment with an increment of 41.01%. Compared with untreated CF/PEEK (Figure 11a) with AA1 plasma treatment samples (Figure 11i), the retained matrix of AA1 was greater, and there was PEEK resin on the surface of the CF. The increase in IFSS can be attributed to the increased mechanical interlocking and chemical bonding. Overall, the trend of IFSS was consistent with the polar/element ratio for strong chemical interaction. However, the increment was not proportional to the polar group contents for the effect of roughness and wettability between CFs and PEEK fibers after plasma treatment. It can be concluded that plasma treatment by Ar–air can significantly increase the interfacial strength between CFs and PEEK fibers and can be applied for the modification of CF/PEEK woven fabrics.

## 4. Conclusions

In this work, the physical and chemical structures of CFs and PEEK fibers before and after plasma treatments were investigated by both surface morphology and surface chemical structure analyses using SEM, AFM, Raman spectra, FTIR, and XPS. In addition, IFSS was tested using the microdroplet test.

SEM results demonstrated the surface morphology of fibers, thus providing a brief explanation of the physical combination and interlock between CFs and PEEK fibers. Improving CF roughness is propitious to the enhancement of its IFSS. The significant properties of CFs and PEEK fibers were studied to examine the effect of plasma over different testing times. In conclusion, plasma treatments were unable to affect the tensile strength of carbon fibers and the thermal properties of PEEK fibers within 10 min in our study. XPS analyses indicated the chemical state of the surface. Plasma treatment was shown to be an effective method for increasing oxygen content and active groups on CFs and PEEK fibers.

Active groups partly revealed the interfacial interaction and adhesion between CF and PEEK during the molding process. To make the work more satisfactory, IFSS values after different plasma treatments were tested, and composites treated with Ar for 1 min and air for 1 min in sequence had the highest IFSS of 59.73 ± 3.88 MPa, with an increment of 41.01%, indicating the effectiveness of plasma modification.

This study offers an in-depth understanding of the intrinsic properties of CFs and PEEK fibers after plasma modification and demonstrates the interfacial strength between them. Further investigations will focus on performing experimental studies on the fabrication of CF/PEEK composites using CF/PEEK hybrid fabrics and comparing properties of CF/PEEK hybrid composites before and after plasma treatment.

## Figures and Tables

**Table 1:** Raman analysis: *R* and surface crystalline size (*L_a_*) of untreated and treated carbon fibers.

Sample	Peak Position		*R* (*I_D_/I_G_*)	*L_a_* (Å)
	D-Band	G-Band		
**Untreated CF**	1364.19	1572.40	2.64 ± 0.04	16.67
**CF–air**	1383.35	1592.91	2.65 ± 0.06	16.60
**CF–Ar**	1385.62	1592.01	2.66 ± 0.05	16.54
**CF–AA15**	1368.41	1577.59	2.66 ± 0.04	16.54
**CF–AA1**	1382.37	1590.71	2.70 ± 0.05	16.30
**CF–AA5**	1377.20	1588.05	2.70 ± 0.04	16.30

**Table 2:** Tensile strength of CFs after different plasma treatments.

Sample	A	B	σ_0_ (GPa)	Γ(σ_0_)	$\bar{\sigma_{f}}(\mathrm{GPa})$
Untreated CF	−12.59	10.24	2.33	0.9514	3.25
CF–AA15	−8.68	6.84	2.00	0.9330	3.31
CF–AA1	−6.25	4.85	1.62	0.9156	3.32
CF–AA5	−7.34	5.83	1.80	0.9267	3.26

**Table 3:** Assignments of the FTIR peaks of PEEK materials.

Wavenumber (cm^−1^)	Assignment
3051	C=C–H stretch vibration
2912, 2866	–CH2 stretch vibration
1652	C=O stretch in ketone
1580	Skeletal in-plane vibration of aromatic ring
1474, 1407	Aromatic rotations
1300–1050	Diphenyl ether group, C–O–C rotation and stretch
923	Aromatic out-of-plane bending
825, 757	C–H out-of-plane bending substitution patterns

**Table 4:** Surface chemical composition of CFs and PEEK fibers.

Samples	Relative Concentration of Elements (%)			O:C	N:C
	C	O	N		
Untreated CF	85.15	14.85	-	0.17	-
CF–air	61.51	34.65	3.84	0.56	0.06
CF–Ar	75.44	21.85	2.71	0.29	0.04
CF–AA1	59.86	35.77	4.38	0.60	0.07
CF–AA5	53.24	41.82	4.94	0.79	0.09
PEEK	86.13	13.87	-	0.16	-
PEEK–AA1	67.48	31.21	1.31	0.46	0.02

**Table 5:** Deconvolution of C1s and the concentration of relevant functional groups from XPS.

Samples	C1	C2	C3	C4	C5	Polar/Nonpolar	Ratio
	C=C	C–C	C–O	–C=O	O–C=O		
Untreated CF	26.85	45.25	21.02	3.43	3.45	0.39	0.07
CF–air	25.24	31.64	27.92	11.30	3.89	0.76	0.48
CF–Ar	23.00	33.60	22.77	12.96	7.66	0.77	0.25
CF–AA1	22.70	33.20	21.30	7.16	15.65	0.79	0.53
CF–AA5	23.20	42.90	16.44	10.33	7.13	0.51	0.45
PEEK	36.32	13.81	40.73	1.99	7.15	0.99	0.16
PEEK–AA1	29.35	9.41	41.65	9.32	10.28	1.58	0.76

**Table 6:** IFSS of untreated and plasma treated CF/PEEK system.

Sample	IFSS (MPa)	Increment (%)
Untreated	42.36 ± 4.23	-
Air	48.37 ± 2.87	14.19
Ar	47.62 ± 3.25	12.42
AA15	50.87 ± 1.79	20.09
AA1	59.73 ± 3.88	41.01
AA5	47.85 ± 4.51	12.96
